# War in Ukraine: handling of manuscripts from the Russian federation by radiation and environmental biophysics

**DOI:** 10.1007/s00411-022-00980-8

**Published:** 2022-07-16

**Authors:** Andrzej Wojcik, Anna A. Friedl, Werner Rühm

**Affiliations:** 1 MBW Department, Centre for Radiation Protection Research, Stockholm University, Stockholm, Sweden; 2 University Hospital, Ludwig-Maximilians-University LMU Munich, Munich, Germany; 3 Institute for Radiation Medicine, Helmholtz Center Munich, 85764 Neuherberg, Germany

Springer Nature, similarly as other publishing houses, condemns the Russian invasion of Ukraine. We, the Editors of the journal of *Radiation and Environmental Biophysics*, unconditionally support the condemnation and echo the statement of Frank Vrancken Peeters, the Chief Executive Officer of Springer Nature: “Our thoughts are with all those affected by the unfolding situation across the region. We join the call for a ceasefire and the return to peace. Peace is one of the UN’s Sustainable Development Goals and a prerequisite for human progress. As an academic and education publisher dedicated to building bridges of understanding, we are shocked and saddened by an act which is designed to drive people apart” (https://www.springernature.com/de/advancing-discovery/springboard/blog/blogposts-open-research/springer-nature-condemns-russian-invasion/20191448).

A question discussed by journal editors since the invasion is whether manuscripts submitted by scientists affiliated to institutions in the Russian Federation should be considered for publication. For example, Olesia Vashchuk, the head of Ukraine’s Young Scientists Council at the Ukrainian Ministry of Education and Science, wrote in March 2022: “Russian scientists have no moral right to retransmit any messages to the world scientific community” (Else 2022). Besides ethical considerations, the idea is that rejecting manuscripts written by authors affiliated to Russian institutions could contribute to the end of the war by exerting pressure on Russian citizens that in turn press the government. A similar idea of exerting pressure on the government, albeit in a direct way, is used to justify the international economic and political sanctions that are currently applied. However, virtually all publishing houses take the stand that boycotting submissions from Russia runs against the principle of a free exchange of ideas among the non-exclusive international scientific community.

We consulted Springer Nature and received the information that the Publisher rejects the idea of boycotting submissions from the Russian Federation and that this policy is based on the advice of COPE (Committee on Publication Ethics) that reads as follows: “Editorial decisions should not be affected by the origins of the manuscript, including the nationality, ethnicity, political beliefs, race, or religion of the authors. Decisions to edit and publish should not be determined by the policies of governments or other agencies outside of the journal itself, except where a decision might place the journal in violation of applicable law” (https://publicationethics.org/news/clarification-cope-advice-editors-geopolitical-intrusions-editorial-decisions). It appears to us that the COPE statement does not directly apply to the idea of boycotting submissions from scientists affiliated to Russian institutes, because such a boycott would not be directed against individuals but against state organisations. Specifically, it would imply rejecting submissions from people working for these organisations, regardless of nationality, ethnicity, political beliefs, race, or religion. Also, such a boycott would not be prompted by any direct governmental influence on the journal editors. Rather, it would be motivated by the policy of the Russian government towards the international community.

The above considerations demonstrate the complexity of the situation. Which policy will do most good and least harm? We do not know. In any case, in accordance with the policy of the Publisher, we as the Editors of the journal of *Radiation and Environmental Biophysics* will continue to handle submissions of scientific papers from the Russian Federation in the spirit of freedom of science. However, this should not be interpreted in any way as support of the war against Ukraine driven by the government of the Russian Federation.
